# Synergistic tenderization and oxidative stabilization of cooked beef by combined fig latex and sodium phytate

**DOI:** 10.1016/j.fochx.2026.104475

**Published:** 2026-09-18

**Authors:** Yao Zhang, Xia Zhou

**Affiliations:** Jiangsu Agri-animal Husbandry Vocational College, 8 Fenghuang East Road, Taizhou, Jiangsu 225300, China

**Keywords:** Fig latex, Sodium phytate, Beef leg chunks

## Abstract

The effect of combination of fig latex and sodium phytate on the quality and shelf life of cooked beef leg muscles during refrigerated storage was tested. The greatest textural improvement was obtained with the combination of 20 g/kg fig latex and 200 mg/kg sodium phytate, decreasing shear force to 19.15 N compared to 42.05 N for 20 g/kg fig latex treatment. Lipid oxidation (TBARS) was reduced by 33.3% compared to untreated controls (0.24 ± 0.02 vs. 0.36 ± 0.02 μmol/g). These are caused by the proteolysis mediated by ficin and the metal-chelating antioxidant action of phytate. This natural approach effectively improves tenderness and reduces oxidative spoilage under the tested conditions, and maintains sensory acceptability over the 10-day refrigerated storage period without the use of synthetic additives; however, further microbiological evaluation and industrial-scale validation are required to confirm shelf-life extension.

## 1 Introduction

Tenderness of meat is an important parameter, affecting consumer satisfaction and value of the product. However, standard heat treatment usually deteriorates this property. Heat treatment enhances microbiological safety and increases shelf life but results in protein denaturation, moisture loss, and a hardening of the muscle fibre structure, resulting to increased toughness and unattractive colour changes from myoglobin oxidation (Faustman et al., 2010; Shah et al., 2014).

Advances in technologies such as high pressure processing, sous-vide cooking and gamma irradiation have revealed meat tenderising potential. For instance, hot-boned beef texture was enhanced by high-pressure treatment (Morton et al., 2017) and the hardness of beef steaks was reduced by sous-vide mixed with freezing (Kim et al., 2020). However, these solutions are typically costly, difficult or subject to regulations and therefore not easily adopted by small and medium firms. As a result, there is increasing interest in natural, food grade chemical agents that may be easily added to current processing lines.

Two promising additions are sodium phytate and fig latex. Sodium phytate (inositol hexaphosphate) is a strong metal chelator, and is widely present in cereals and legumes. It will bind pro-oxidant ions such as Fe^2+^ and Cu^2+^. This characteristic efficiently suppresses lipid peroxidation and protein oxidation in meat products, and also stabilises the colour and improves the water holding capacity (Lee et al., 1998; Liu et al., 2010). Ficin is a cysteine protease that destroys myofibrillar and connective tissue proteins. Fig latex is obtained from *Ficus carica* L. and contains polyphenols, flavonoids and organic acids that have antioxidant and antibacterial effects (Baeyens-Volant et al., 2015; Solomon et al., 2006). These bioactive elements together contribute to softness, inhibit oxidative rancidity and inhibit microbial growth (Falowo et al., 2014; Fathy et al., 2023).

Despite the individual benefits of each addition, the cumulative effect on cooked beef has not been rigorously investigated. While plant proteases (e.g., papain, bromelain) and sodium phytate have been studied separately for meat tenderization or antioxidant protection, their combined application and potential synergistic interaction in cooked beef remain unexplored; the specific scientific gap lies in the lack of systematic optimization of dual dosage and evaluation of their cooperative effects on texture and oxidative stability during chilled storage. We propose that sodium phytate could enhance the effectiveness of fig latex as an enzymatic tenderiser by reducing metal-catalyzed protein crosslinking, and that phenolic chemicals in latex may enhance the effectiveness of phytate as an antioxidant. Thus, the main novelty of this study is not the use of either agent alone, but the demonstration of their synergistic interaction through optimized formulation and the integrated assessment of tenderness, lipid oxidation, and sensory acceptability during refrigerated storage, distinguishing it from previous single-agent approaches. The objective of the present study was to identify the optimum levels of fig latex and sodium phytate for tenderness, inhibition of lipid oxidation, sensory qualities and refrigerated shelf life of cooked beef leg slices, with the aim of providing a realistic and natural alternative for meat processors seeking to replace synthetic preservatives with clean-label solutions.

## 2 Materials and methods

### 2.1 Raw materials and reagents

Fresh beef hind limb muscles (*M. semimembranosus* and *M. biceps* femoris) were sourced from Yunrun Group (Nanjing, China). The animals were Chinese Yellow cattle aged 24–30 months. Carcasses were chilled at 0–4 °C for 48 h post-mortem to complete rigor, reflecting standard industrial practice. Food-grade sodium phytate (≥99% purity) was purchased from WanJiWei Biological Engineering Co., Ltd. (Laiyang, Shandong, China).

### 2.2 Fig latex collection and characterization

Latex was harvested from unripe *F. carica* L. fruits cultivated in Jiangsu Province by manual incision. The exudate was immediately filtered through double-layered gauze and stored at −20 °C. Before use, it was thawed and diluted with distilled water. Protein quantification followed the Bradford (1976), and ficin proteolytic activity was assayed using casein as substrate (Devaraj et al., 2008). Total phenolics were measured with Folin-Ciocalteu reagent (Singleton et al., 1999), and total flavonoids via aluminum chloride colorimetry (Zhishen et al., 1999). Vitamin C content was determined by 2,6-dichlorophenolindophenol titration (AOAC, 2005). Organic acids (citric, malic) were analysed by HPLC (Kwon et al., 2023). All latex employed in this study originated from a single harvest batch and the same cultivation area to reduce natural variability. We acknowledge that fig latex is inherently variable; future investigations should evaluate seasonal fluctuations, batch-to-batch reproducibility, and storage stability to support industrial standardization.

### 2.3 Optimization of fig latex dosage

To ensure biological replication, beef hind limb muscles (M. semimembranosus and M. biceps femoris) were obtained from six independent Chinese Yellow cattle (24–30 months old, one hind leg per animal). Muscles were trimmed of visible fat and connective tissue, and cut into uniform cubes (5 × 5 × 5 cm). From each of the six animals, seven cubes were randomly assigned to seven fig latex concentrations: 0, 5, 10, 15, 20, 25, or 30 g/kg (one cube per concentration per animal, resulting in six biological replicates per concentration, *n* = 6). Each cube was immersed in a 2:1 (*w*/*v*) water-additive mixture at 4 ± 1 °C for 120 min with gentle agitation (50 rpm). After marination, cubes were boiled for 1 h, cooled to 25 °C, and shear force was measured using a TA.XTplus Texture Analyzer fitted with a Warner-Bratzler blade (crosshead speed 200 mm/min). One-way ANOVA with Tukey's HSD test (*P* < 0.05) identified 20 g/kg as the optimal fig latex concentration.

### 2.4 Optimization of sodium phytate concentration

Using the optimal fig latex concentration (20 g/kg) determined above, beef cubes from the same six animals were treated with increasing concentrations of sodium phytate (0, 50, 100, 150, 200, 250, 300 mg/kg) in double-distilled water containing 20 g/kg fig latex. For each concentration, six cubes (one from each animal) were immersed at 4 °C for 120 min, then boiled for 1 h, cooled, and subjected to shear force measurement as described in Section 2.3. The concentration yielding the lowest shear force (200 mg/kg) was selected as optimal.

### 2.5 Shear force measurement

Shear force was determined following the Warner-Bratzler square shear method (Silva et al., 2015). From each cooked cube, three 1 × 1 × 1 cm sub-samples were cut parallel to the muscle fibre orientation and sheared perpendicularly using the texture analyzer. The average value of the three measurements from each cube was used as one biological replicate. Six biological replicates (*n* = 6, one per animal) per treatment were analysed.

### 2.6 Sensory assessment

Cooked beef cubes (1.5 cm edge) from all sodium phytate treatment groups were steamed for 3 min and presented to a trained panel of 24 evaluators (12 male, 12 female; age 25–50 years) across three randomized sessions (24 h apart). Panelists were trained in two sessions using commercial beef references. Evaluations were conducted in individual booths under controlled lighting and temperature (22 ± 2 °C), with water rinsing and cracker consumption between samples. The rejection threshold (score = 5.0) was based on prior literature (Sales et al., 2020; Verbeke et al., 2010). Each sample was coded with a three-digit number and served in random order. Panelists rated overall liking on a 9-point hedonic scale (1 = extremely dislike, 9 = extremely like). The study received ethical approval from Jiangsu Ocean University's Institutional Review Board, and all participants gave written informed consent.

### 2.7 Lipid oxidation assessment

Lipid peroxidation was quantified using the TBARS assay (Raharjo et al., 1992). For each treatment, six cooked cubes (one from each animal) were individually analysed. Results were expressed as mg malondialdehyde (MDA) equivalents per kg sample, calibrated against a 1,1,3,3-tetraethoxypropane standard curve. Data are presented as mean ± standard deviation of six biological replicates (*n* = 6).

### 2.8 Storage stability trial

Beef chunks from the six animals were treated with the optimal combination (20 g/kg fig latex + 200 mg/kg sodium phytate) under the same marination conditions (4 °C, 180 min). After boiling (1 h) and cooling, samples were vacuum-packed and stored at 4 ± 0.5 °C for 10 days. On days 0, 2, 4, 6, 8, and 10, six independently treated samples (one from each animal) were withdrawn for shear force, TBARS, and sensory analyses. Untreated controls (six samples from the same six animals) were evaluated in parallel on days 0, 2, 4, and 6; control sampling was discontinued after day 6 because the sensory scores of untreated samples fell below the consumer rejection threshold (score = 5.0).

### 2.9 Statistical analysis

The experiment followed a completely randomized design with six independent biological replicates per treatment (*n* = 6; each replicate originated from a different animal). Normality and homoscedasticity were verified using Shapiro-Wilk and Levene's tests. Sensory data were analysed using a linear mixed-effects model: Sensory Score ∼ Treatment + (1 | Panelist) + (1 | Session) + (1 | Panelist:Session), with treatment as fixed effect and panelist, session, and their interaction as random effects. Model fit improvement was confirmed by likelihood ratio test (χ^2^ = 15.3, *P* < 0.001). For single-factor optimization, shear force and TBARS data were analysed by one-way ANOVA; for combined treatments, two-way ANOVA with interaction term (fig latex × sodium phytate) was used to test synergy. Tukey's HSD test (*P* < 0.05) was applied for post-hoc comparisons using SPSS v26.0. Outliers exceeding ±3 standard deviations were removed, and sensitivity analyses confirmed robustness. Effect sizes (partial η^2^ > 0.14) indicated practical significance.

## 3 Results and discussion

### 3.1 Composition profile of fig latex

Table1 summarises the bioactive compounds present in fig latex. Ficin represented 12.31% of the total protein and showed a specific proteolytic activity of 85.62 U/mg. Total phenolics were 6.18 ± 0.27 mg GAE/g, flavonoids 2.82 ± 0.19 mg RE/g and vitamin C 1.49 ± 0.11 mg/g. Besides organic acids (citric 0.79%, malic 0.61%), tannins, saponins, terpenoids and coumarins were also found. In this complicated mixture, myofibrillar and connective tissue proteins are degraded by ficin, and radical scavengers are phenolic compounds and vitamin C (Baeyens-Volant et al., 2015; Zhou et al., 2023). The presence of organic acids and tannins may also contribute to antibacterial properties, however this was not experimentally investigated.

**Table 1:** Active components and functions of fig latex. Data are from a single harvest batch; analyses were performed in triplicate technical replicates, with coefficients of variation <5% for all reported values.

Component	Function	ContentorNotes
Ficin (Proteolytic Enzyme)	Tenderization via protein hydrolysis	∼12.31% of total protein; activity: 85.62 U/mg
Polyphenols	Antioxidant activity via free radical scavenging	6.18 ± 0.27 mg GAE/g latex
Flavonoids	Antioxidant and anti-inflammatory effects	2.82 ± 0.19 mg RE/g latex
Vitamin C (Ascorbic Acid)	Redox activity, enhances antioxidant system	1.49 ± 0.11 mg/g latex
Organic Acids	Antimicrobial activity, pH reduction	Citric acid: 0.79 ± 0.03%; Malic acid: 0.61 ± 0.02% (*w*/w)
Tannins	Astringent, antimicrobial, antioxidant	0.71 ± 0.05% w/w
Saponins	Surface-active, antimicrobial and antioxidant	0.45 ± 0.03% w/w
Terpenoids	Bioactive modulation, antioxidant and antimicrobial	0.32 ± 0.02% w/w
Coumarins	Antimicrobial and enzyme-modulating properties	0.15 ± 0.01% w/w

### 3.2 Synergy in reduction of shear force

Fig.1 exhibits the effect of fig latex and sodium phytate on shear force of cooked beef leg chunks. All samples were marinated under the same conditions (4 °C for 180 min). The shear force decreased gradually with increase of fig latex content as shown in Fig. 1a. The minimum value 42.05 N was obtained at 20 g/kg. Higher concentrations, 25 to 30 g/kg did not enhance tenderness further.

**Fig. 1:**
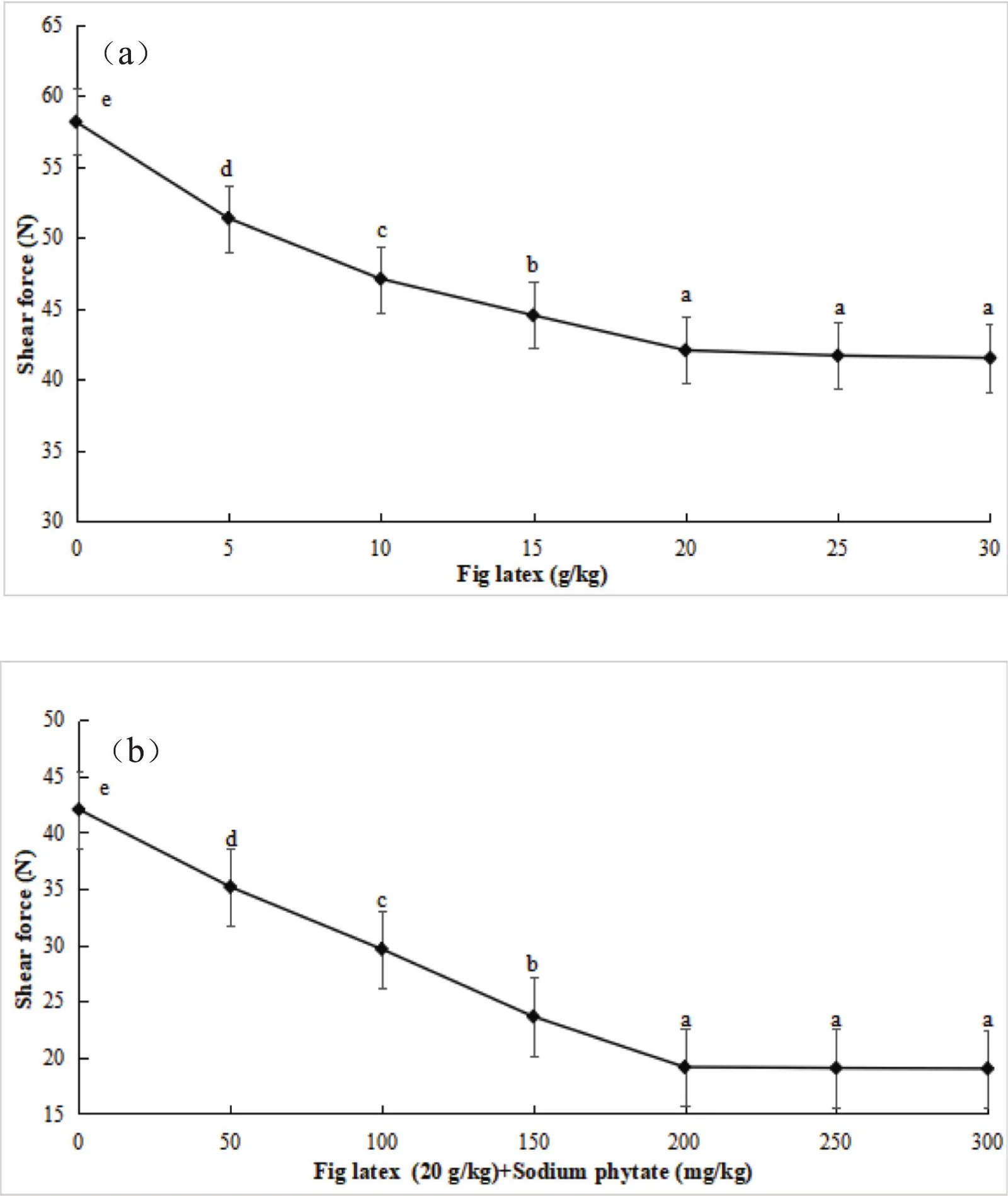
The effects of the concentrations of fig latex and sodium phytate on shear force of the cooked beef leg chunks. Data are presented as mean ± standard deviation (*n* = 6 biological replicates, each from a different animal). Two-way ANOVA revealed a significant synergistic interaction between fig latex and sodium phytate (*P* < 0.05).

The addition of sodium phytate to the base treatment with 20 g/kg fig latex resulted in an additional reduction of shear force (Fig. 1b). The minimum shear force was 19.15 N (200 mg/kg sodium phytate). This value was clearly lower than that of fig latex alone (42.05 N). It was also substantially lower than the untreated control, which was >36 N. Two-way ANOVA demonstrated a strong synergistic interaction between the two additions (*P* < 0.05).

The tenderising effect of fig latex is due to the proteolytic activity of ficin, a cysteine protease present in the latex. The enzyme degrades myofibrillar and connective tissue proteins (Baeyens-Volant et al., 2015; Devaraj et al., 2008). At the same time, sodium phytate functions by chelating pro-oxidant metal ions, e.g. Fe^2+^ and Cu^2+^. Phytate-mediated metal chelation can effectively mitigate metal-catalyzed protein cross-linking during thermal processing (Kumar et al., 2010; Lee et al., 1998; Liu et al., 2010), which may further account for the additional reduction in shear force observed following the combined treatment of sodium phytate and fig latex. Nevertheless, the above explanation remains speculative, as the present study did not conduct direct quantification of protein cross-linking or structural modification. Accordingly, the tenderness-enhancing effect of sodium phytate should be recognised as a preliminary finding, and its underlying mechanism requires further systematic verification. Together these two methods create a more pronounced tenderising impact than any ingredient alone. The synergistic bioactivity observed herein is presumed to arise from the complementary interplay between ficin-induced proteolysis and phytate-driven metal chelation; however, this mechanistic interpretation is preliminarily inferred from shear force data and lacks direct molecular evidence. Further corroborative investigations—including SDS-PAGE analysis, quantification of protein carbonyl and sulfhydryl groups, collagen solubility determination, and microstructural observation—are essential to systematically validate the underlying mechanisms.

### 3.3 Optimum marination time and texture

Fig. 2 shows the shear force and overall liking of the consumers as a function of marination time (0–180 min) at 4 °C with the ideal single concentration of fig latex (20 g/kg). The shear force gradually dropped from 58.16 N at time zero to 24.25 N after 120 min. Consumer liking scores presented a bell-shaped response with the maximum hedonic score (8.8/9.0) at the shear force of 24.25 N (120 min). Shorter marination periods were associated with lower liking scores and harder meat. Notably, when sodium phytate was added together with 20 g/kg fig latex at the same marination time (120 min), the shear force was further reduced to 19.15 N (Fig. 1b). This additional tenderisation beyond the optimal single-agent level resulted in a decline in liking scores (to 5.9/8.8), as evaluated in separate sensory tests using the same panel and protocol. This means that over-tenderisation (shear force <24 N) generates a pasty or mushy texture that is not so good in some culinary traditions. Moderate chewiness (24–30 N) is preferable in braised Chinese beef (Sales et al., 2020; Verbeke et al., 2010), but ultra-tender beef (<20 N, prevalent in Western markets) is undesirable. This observation should be interpreted cautiously, as consumer preference for tenderness varies across cultural contexts; the present results reflect a Chinese panel and may not generalize to other populations. The radical scavenging activity of fig latex is also due to phenolic chemicals, flavonoids and vitamin C (Solomon et al., 2006; Zhou et al., 2023), however the main texture-modifying effect is due to ficin. Meat producers can therefore adjust the level of sodium phytate to suit market preferences: lower levels for Asian consumers who seek moderate firmness and higher levels for Western markets where extreme softness is preferred.

**Fig. 2:**
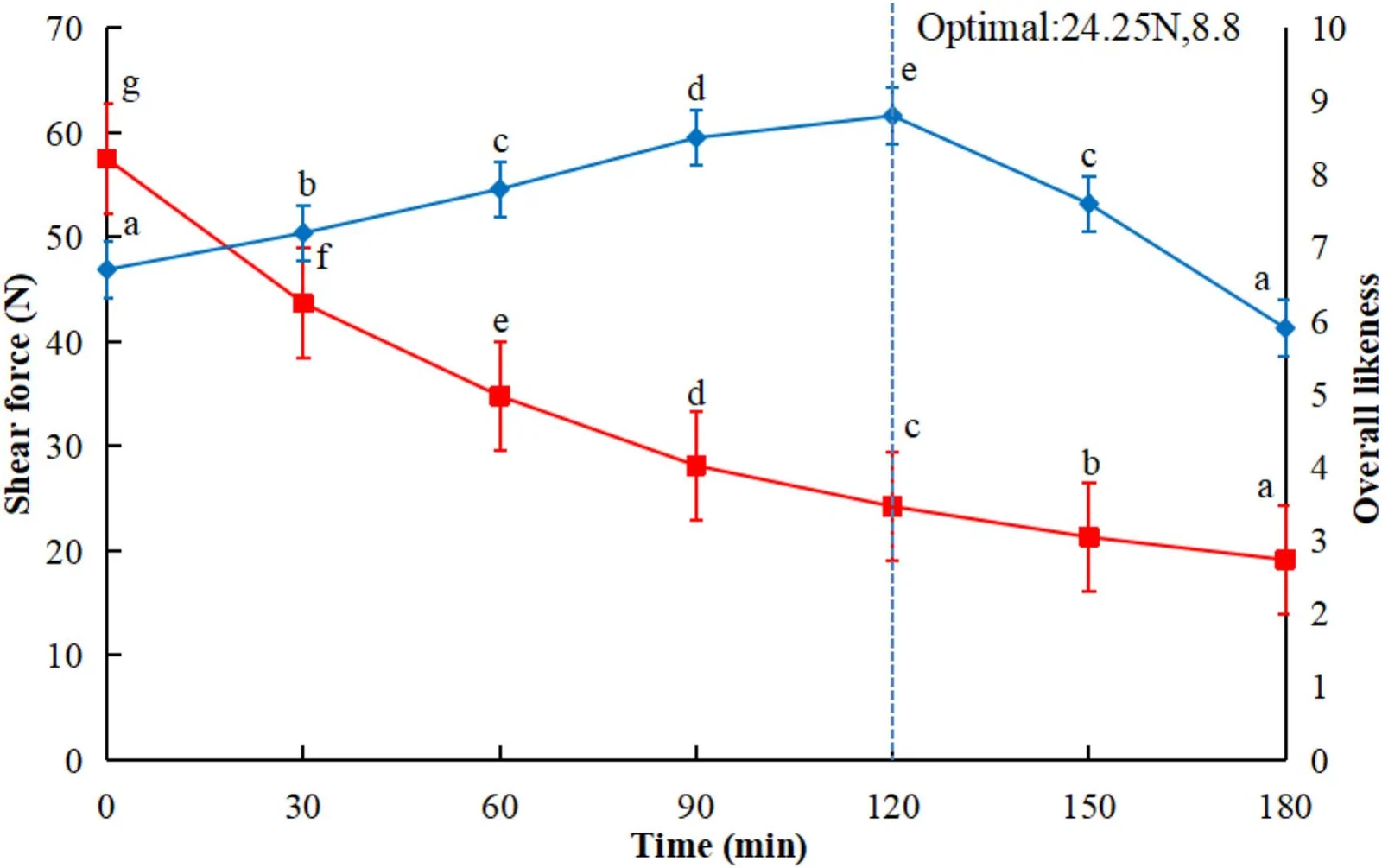
Effect of pickling time on shear force (■, left axis) and overall likeness (◆, right axis) of cooked beef leg chunks. The highest liking score (8.8) was observed at 120 min, corresponding to a shear force of 24.25 N. Data are presented as mean ± standard deviation (*n* = 6 biological replicates, each from a different animal). Different letters indicate significant differences among marination times for shear force (uppercase) and sensory scores (lowercase) (*P* < 0.05, Tukey's HSD).

### 3.4 Lipid peroxidation inhibition

The lipid oxidation during heat processing was studied by measuring TBARS values. The combination of 20 g/kg fig latex and 200 mg/kg sodium phytate decreased TBARS to 0.24 ± 0.02 μmol/g, a 33.3% drop compared with the untreated controls (0.36 ± 0.02 μmol/g). Two-way ANOVA indicated a significant interaction (*P* < 0.05) between the two additives. Sodium phytate alone dose-dependently decreased TBARS from 0.35 μmol/g (0 mg/kg) to 0.24 μmol/g (300 mg/kg) without further improvement at higher dosages. This impact is due to chelation of Fe^2+^ and Cu^2+^ which inhibits Fenton-driven radical production (Huang et al., 2018; Kumar et al., 2010). Polyphenols, flavonoids and vitamin C in latex fig are chain-breaking antioxidants and free radical scavengers (Solomon et al., 2006; Zhou et al., 2023). The two work well together in complementary methods to ensure effective oxidative protection, which maintains flavour and colour. Nevertheless, the current experimental setup fails to quantitatively differentiate the primary metal chelation exerted by sodium phytate from the secondary radical-scavenging activity of fig polyphenols. According to oxidation kinetic characteristics, phytate-mediated metal chelation is speculated to predominate in the early oxidation initiation stage via the inhibition of Fenton reactions. By contrast, phenolic constituents in fig latex function as a vital auxiliary factor to terminate radical chain propagation during the late storage phase. Further targeted experiments, including sequential reagent addition and selective functional masking, are therefore necessary to decouple and clarify the respective synergistic contributions of these two active components.

### 3.5 Stability on long-term storage

Treated samples exhibited significantly lower shear force and TBARS readings than controls throughout 10 days of refrigerated storage (4 °C) (Fig. 3). On day 10, TBARS in the dual treated group was 0.28 ± 0.03 μmol/g (controls were not measured beyond day 6 as their sensory scores had already fallen below the rejection threshold). Sensory scores (Fig. 4) for treated samples were above the consumer rejection threshold of 5.0 for the 10-day period while controls were below this limit by day 6. The continued quality is credited to the structural modifications imparted by ficin during marination (Baeyens-Volant et al., 2015; Devaraj et al., 2008), metal chelating action of phytate (Kumar et al., 2010; Lee et al., 1998) and radical scavenging capacity (Solomon et al., 2006; Zhou et al., 2023) of phenolics in fig latex. While these attributions are consistent with the observed quality changes, direct molecular evidence—such as protein degradation patterns, oxidation markers, or structural analyses—was not obtained in the present study and remains a direction for future investigation. Notably, since ficin is a heat-labile cysteine protease, the enzyme is likely inactivated during the boiling step; therefore, the maintenance of tenderness during storage probably reflects pre-existing proteolytic modifications rather than continued enzymatic activity. These findings show that the natural dual treatment can effectively delay oxidative rancidity, retain suppleness and maintain sensory acceptability for up to 10 days without the use of synthetic preservatives (Falowo et al., 2014). Future studies should investigate longer storage periods, freezing conditions, and cost effective scaling for industrial usage. Recent studies have reported tenderising effects of plant-derived proteases and antioxidants in meat (Falowo et al., 2014; Shah et al., 2014). Our dual strategy offers combined benefits, though direct comparisons are limited. We acknowledge the short storage period, lack of microbiological and molecular data, and laboratory-scale design as limitations.

**Fig. 3:**
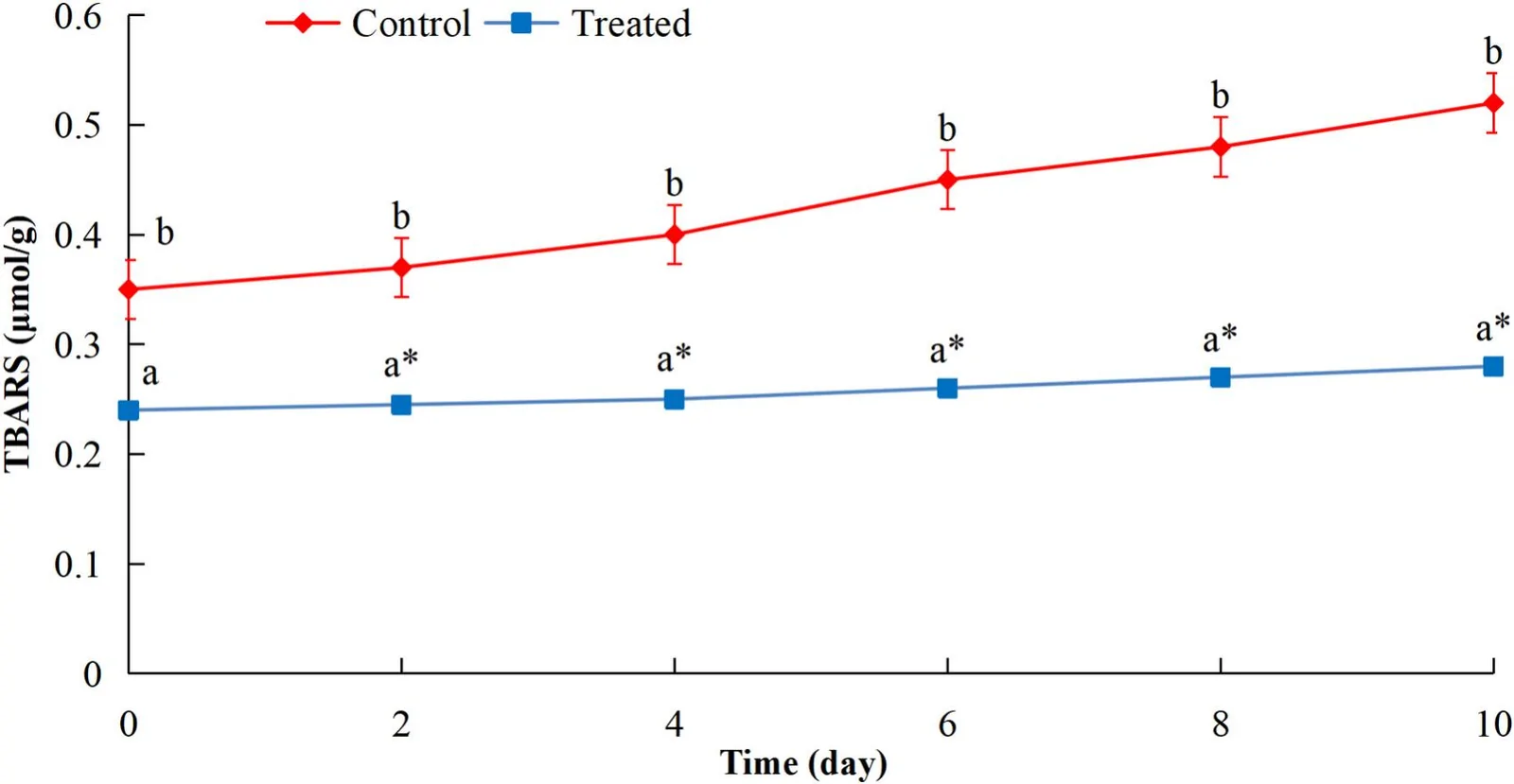
Changes in TBARS values of cooked beef leg chunks during 10 days of refrigerated storage at 4 °C. Control: untreated samples; Treated: samples treated with 20 g/kg fig latex and 200 mg/kg sodium phytate. Data are presented as mean ± standard deviation (*n* = 6 biological replicates, each from a different animal). Significantly different from control at the same time point (*P* < 0.05); different letters indicate significant differences among storage times within the same treatment group (*P* < 0.05, Tukey's HSD).

**Fig. 4:**
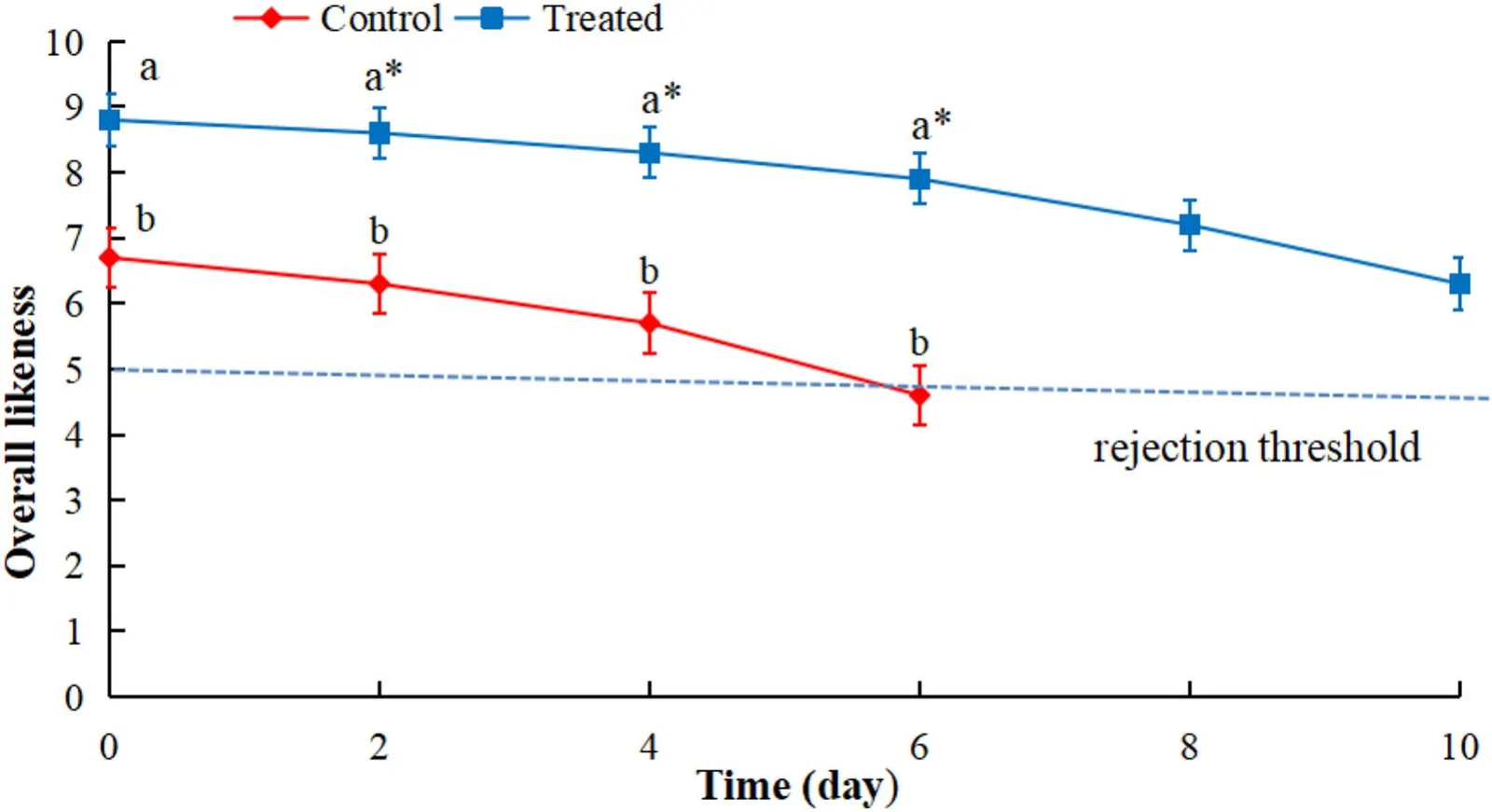
Changes in overall acceptability scores of cooked beef leg chunks during 10 days of refrigerated storage at 4 °C. Control: untreated samples; Treated: samples treated with 20 g/kg fig latex and 200 mg/kg sodium phytate. Data are presented as mean ± standard deviation (*n* = 24 panelists). The dashed line indicates the consumer rejection threshold (score = 5.0). Significantly different from control at the same time point (*P* < 0.05); different letters indicate significant differences among storage times within the same treatment group (*P* < 0.05, Tukey's HSD).

## 4 Conclusions

The combination of fig latex (20 g/kg) and sodium phytate (200 mg/kg) showed a synergistic effect in enhancing softness (shear force decreased to 19.15 N), inhibiting lipid oxidation (TBARS reduced by 33.3%) and maintaining sensory acceptability for up to 10 days under refrigerated storage. The effects were preliminarily attributed to proteolysis by ficin and the potential metal chelating characteristics of phytate; however, direct molecular evidence for these mechanisms was not obtained in this study. This clean-label strategy shows promise as a potential alternative to synthetic preservatives; however, actual shelf-life extension requires further microbiological validation, and practical application requires evaluation of raw material standardization, regulatory approval, production costs, batch consistency, allergenicity of fig latex, and industrial compatibility. Longer storage times, freezing conditions and cost benefit evaluations should also be addressed in future research.

## CRediT authorship contribution statement

**Yao Zhang:** Writing – original draft, Visualization, Validation, Software, Investigation, Formal analysis, Data curation. **Xia Zhou:** Writing – review & editing, Supervision, Resources, Project administration, Methodology, Funding acquisition, Conceptualization.

## Declaration of competing interest

The authors declare that they have no known competing financial interests or personal relationships that could have appeared to influence the work reported in this paper.

## Data Availability

The authors do not have permission to share data.
